# Supplementary material for: Ultra-narrow-band near-infrared thermal exciton radiation in intrinsic one-dimensional semiconductors
Source: Nat Commun. 2018 Aug 7;9:3144. doi: 10.1038/s41467-018-05598-3 (PMC6081476; doi:10.1038/s41467-018-05598-3)
Supplement: Supplementary file 1 — Supplementary Information [file 41467_2018_5598_MOESM1_ESM.pdf]

SUPPLEMENTARY INFORMATION

Ultra-narrow-band near-infrared thermal exciton radiation in intrinsic one-dimensional semiconductors

Nishihara *et.al.*

Supplementary Note 1: Design of high-efficiency narrow-band thermal emitter

At 2100 K, the total emission power in the range 0.8–1.9 eV was experimentally determined as ~5 pW by assuming that radiation from an exciton dipole is collinear to the nanotube axis. The energy conversion efficiency was evaluated as ~5×10^{-6} using the absorbed power of ~1 μW estimated from the cw laser intensity (227 kW cm^{-2}), the excitation spot size (2 μm), and the typical absorption cross section per carbon atom (~10^{-17} cm^2)[1]. The current results indicate that the energy conversion efficiency of the thermal exciton radiation was quite small and that most of the thermal energy is dissipated through heat conduction along the tube axis under the current conditions because of the high thermal conductivity of individual nanotubes[2]. It appears that the efficiency is too low, but it is predicted that this can be drastically enhanced by optimizing the device structure to minimize energy dissipation through direct thermal conduction. A possible strategy is to drastically reduce the thermal conductivity (<~1/1000) by fabricating a membrane consisting of structure-controlled individual semiconducting nanotubes, where the number of nanotube–nanotube point contacts ensures very low thermal conductance[3].

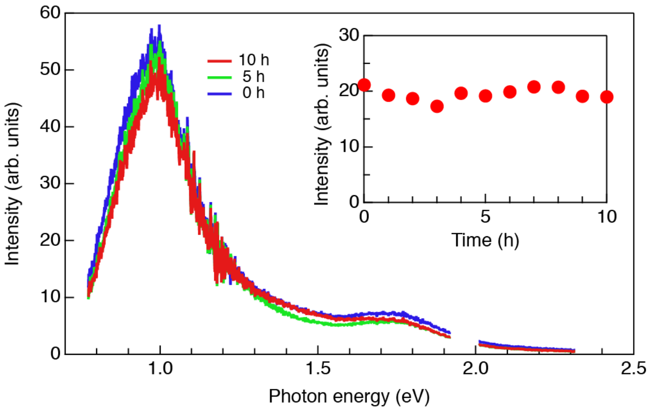

Supplementary Figure 1 | Temporal evolution of the thermal exciton radiation spectra. The light emission spectra of the (15,11) semiconducting nanotube at ~2000 K measured at hourly intervals. Representative spectra at 0 h (blue), 5 h (green), and 10 h (red) are shown. The continuous-wave laser for heating was kept focused on the same region of the nanotube during the experiment. The inset shows the integrated radiation intensities over 0.8–2.3 eV as a function of time.

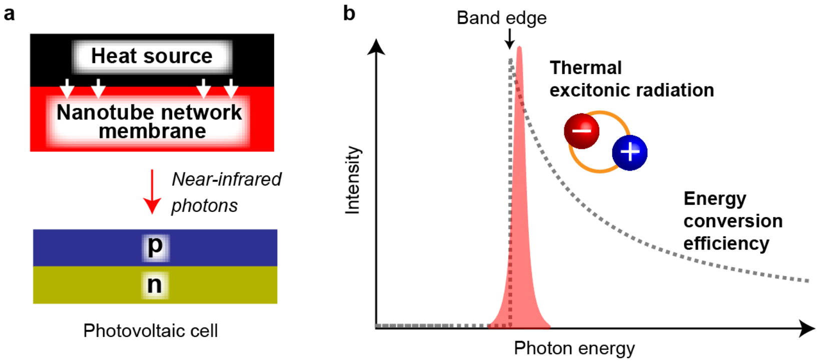

Supplementary Figure 2 | Concept of efficient thermophotovoltaic system. a, Schematic of a thermophotovoltaic system with a nanotube network membrane. The nanotube membrane converts heat to near-infrared photons through the thermal generation of excitons. The input of near-infrared photons to a photovoltaic cell generates electricity. **b,** Schematic of a narrow-band thermal exciton radiation spectrum with an energy conversion efficiency curve of a typical photovoltaic cell. When a photovoltaic cell absorbs a photon with the energy above the bandgap, a single pair of an electron and a hole is generated regardless of the photon energy. Thus, the energy conversion efficiency decreases as the photon energy increases.

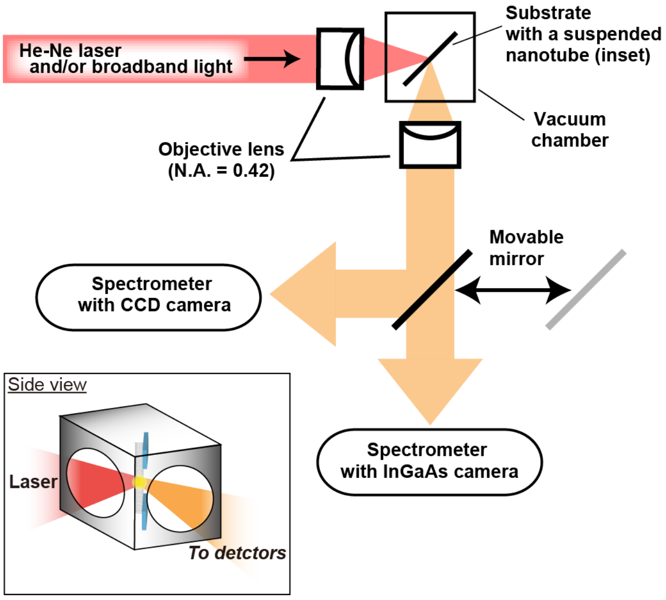

Supplementary Figure 3 | Schematic of dark-field spectroscopy of suspended nanotubes. Movable mirror switches the detection optical paths to the charge-coupled device (CCD) camera (1.2–2.8 eV) or to the indium-gallium-arsenide (InGaAs) camera (0.8–1.4 eV). The inset shows the side view of the vacuum chamber.

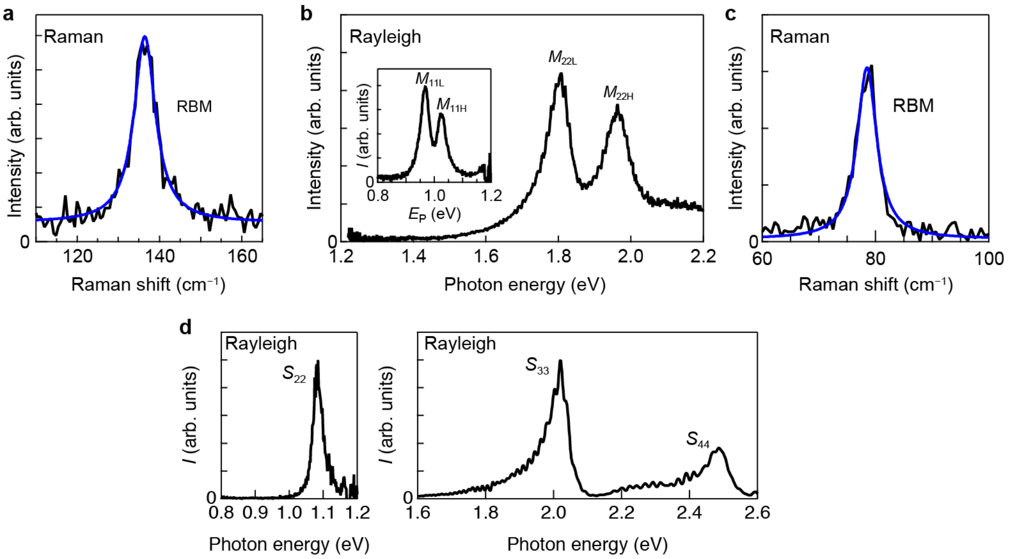

Supplementary Figure 4 | Characterization of individual nanotubes. a, The Raman spectrum of the (18,8) semiconducting nanotube around the radial breathing mode (RBM). **b, c,** Rayleigh (**b**) and Raman (**c**) spectra of the (30,12) metallic nanotube. The blue curves in **a** and **c** are the fits to a Lorentzian function. **d,** Rayleigh spectra of the (15,11) semiconducting nanotube.

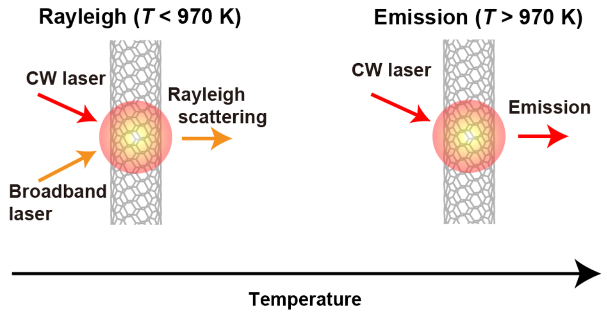

Supplementary Figure 5 | Schematics of the light scattering and emission measurements. The optical susceptibility were probed by means of Rayleigh scattering (≤970 K) and thermally driven radiation (≥970 K). The broadband light and the helium–neon continues-wave (CW) laser were used for Rayleigh spectroscopy, and for heating and Raman spectroscopy, respectively.

Supplementary References

1. Berciaud, S., Cognet, L. & Lounis, B. Luminescence decay and the absorption cross section of individual single-walled carbon nanotubes. *Phys. Rev. Lett.* **101**, 077402 (2008).
2. Jorio, A., Dresselhaus, M. S. & Dresselhaus, G. *Carbon Nanotubes: Advanced Topics in Synthesis, Structure, Properties and Applications*. (Springer, 2008).
3. Prasher, R. S. *et al.* Turning carbon nanotubes from exceptional heat conductors into insulators. *Phys. Rev. Lett.* **102**, 105901 (2009).
